# Smartphone-Enabled Fluorescence and Colorimetric Platform for the On-Site Detection of Hg^2+^ and Cl^−^ Based on the Au/Cu/Ti_3_C_2_ Nanosheets

**DOI:** 10.3390/molecules28145355

**Published:** 2023-07-12

**Authors:** Keyan Chen, Shiqi Fu, Chenyu Jin, Fan Guo, Yu He, Qi Ren, Xuesheng Wang

**Affiliations:** 1 School of Public Health, North China University of Science and Technology, Tangshan 063210, China; kychen1107@163.com (K.C.); fushiqi17_f@163.com (S.F.); jincy2021@126.com (C.J.); gf0518@126.com (F.G.); 2 Ministry of Education Key Laboratory for the Synthesis and Application of Organic Functional Molecules, College of Chemistry and Chemical Engineering, Hubei University, Wuhan 430062, China; heyu@hubu.edu.cn

**Keywords:** Au/Cu/Ti_3_C_2_ NS, Au–Hg alloy, oxidase-like activity, smartphone, colorimetric detection

## Abstract

Smartphone-assisted fluorescence and colorimetric methods for the on-site detection of Hg^2+^ and Cl^−^ were established based on the oxidase-like activity of the Au–Hg alloy on the surface of Au/Cu/Ti_3_C_2_ NSs. The Au nanoparticles (NPs) were constructed via in-situ growth on the surface of Cu/Ti_3_C_2_ NSs and characterized by different characterization techniques. After the addition of Hg^2+^, the formation of Hg–Au alloys could promote the oxidization of o-phenylenediamine (OPD) to generate a new fluorescence emission peak of 2,3-diaminopenazine (ADP) at 570 nm. Therefore, a turn-on fluorescence method for the detection of Hg^2+^ was established. As the addition of Cl^−^ can influence the fluorescence of ADP, the fluorescence intensity was constantly quenched to achieve the continuous quantitative detection of Cl^−^. Therefore, a turn-off fluorescence method for the detection of Cl^−^ was established. This method had good linear ranges for the detection of Hg^2+^ and Cl^−^ in 8.0–200.0 nM and 5.0–350.0 µM, with a detection limit of 0.8 nM and 27 nM, respectively. Depending on the color change with the detection of Hg^2+^ and Cl^−^, a convenient on-site colorimetric method for an analysis of Hg^2+^ and Cl^−^ was achieved by using digital images combined with smartphones (color recognizers). The digital picture sensor could analyze RGB values in concentrations of Hg^2+^ or Cl^−^ via a smartphone app. In summary, the proposed Au/Cu/Ti_3_C_2_ NSs-based method provided a novel and more comprehensive application for environmental monitoring.

## 1. Introduction

Many anions and cations often have a significant influence on physiological functions and the environmental system. For example, Cl^−^ is a common anion with essential physiological functions which takes part in many important biological processes, such as the regulation of cell volume, membrane potential, and intracellular pH. However, excess Cl^−^ can cause severe dehydration and even plant death, as well as health issues such as high blood pressure and various heart diseases [1,2,3]. Hg^2+^ as global environmental pollutants were found in the air, soil, and water. Inorganic mercury (Hg^2+^) can transform into neurotoxin methylmercury (MeHg) through bacterial conversion within water bodies, which can damage the human brain, lungs, and central nervous system via the food chain [4,5,6,7]. Therefore, the detection of trace Cl^−^ and Hg^2+^ is particularly urgent and necessary for disease diagnosis, environmental monitoring, and food safety evaluation [8,9,10,11,12,13]. Various conventional methods have been developed for the detection of Hg^2+^ and Cl^−^, including inductively coupled plasma mass spectrometry (ICP-MS), energy dispersive X-ray spectroscopy (EDX), and ion chromatography [14,15,16,17,18,19]. Despite the significant accuracy and precision of the above methods, they suffer from limitations including expensive equipment, tedious sample preparation, and on-site detection. Therefore, it is necessary to look for facile, portable, and cost-effective strategies for the detection of Cl^−^ and Hg^2+^.

The colorimetric method has attracted the most attention due to its simple readout and obvious color change which can be observed by the naked eye [20,21]. There is a great demand to achieve a high-sensitivity colorimetric method which can greatly expand its applications field [22]. Recently, the nanozyme-based colorimetric method was used for the detection of ion at the nmol/L level, which could be equivalent to the analytical results from expensive equipment. Zhou et al. developed the colorimetric detection of Hg^2+^ by Au nanoparticles formed with the H_2_O_2_ reduction of HAuCl_4_ [23]. Logan et al. demonstrated amalgamated Au-nanoalloys with enhanced catalytical activity for the colorimetric detection of Hg^2+^ in seawater samples [24]. Wang et al. developed a single-nanozyme colorimetric array based on target-induced differential surface passivation for the quantification and discrimination of Cl^−^, Br^−^, and I^−^ [25]. However, Cl^−^ and Hg^2+^ usually coexist in the practical clinical or environmental matrix, with only a few works reported on colorimetric methods for the continuous monitoring of Cl^−^ and Hg^2+^. Yan et al. proposed an efficient strategy for the visual detection and removal of toxic Cl^−^ and Hg^2+^ based on the *β*-Cyclodextrin and graphene oxide co-strengthened AgRu bimetal mesoporous nanozyme [26]. Unfortunately, this method accelerated the oxidation of colorless 3,3′,5,5′-tetramethylbenzidine (TMB) with H_2_O_2_ in blue oxTMB. The instability of H_2_O_2_ could interfere with the accuracy of the corresponding sensing method. Therefore, it is still a challenge to explore the colorimetric methods for the continuous monitoring of Cl^−^ and Hg^2+^ based on oxidase-like nanozymes which catalyzed the substrate without H_2_O_2_.

As a new two-dimensional material, Ti_3_C_2_ nanosheets (NSs) as the most promising MXene material derived from the MAX phase have intriguing metallic conductivity, high stability, a large surface area and ease of functionalization, as well as biocompatibility, which has wide applications in the fields of biosensing, catalysis, energy, and nanomedicine [27,28,29,30,31,32]. MXene cannot be directly synthesized from M and X elements for their thermodynamic metastability. The employment of the HF etching strategy is generally used for the fabrication of MXene, although HF acid is severely corrosive and toxic. Therefore, it is very important to develop a fluorine-free method for MXene preparation for its applications. Recently, Ti_3_C_2_ NSs attracted wide attention for its enzyme-like activity. Chen et al. prepared nitrogen-sulfur-doped Ti_3_C_2_ NSs with peroxidase-like activity and an electrochemical ability for the quantitative detection of uric acid [33]. Wu et al. synthesized histidine-modified Ti_3_C_2_ NSs, which can simulate the catalytic performance of peroxidase for a colorimetric paper-based sensor of glucose [34]. But until now, reports about the Ti_3_C_2_ NSs-based nanozyme with oxidase-like activity as well as nanozyme-based sensing are limited.

**Figure 1:**
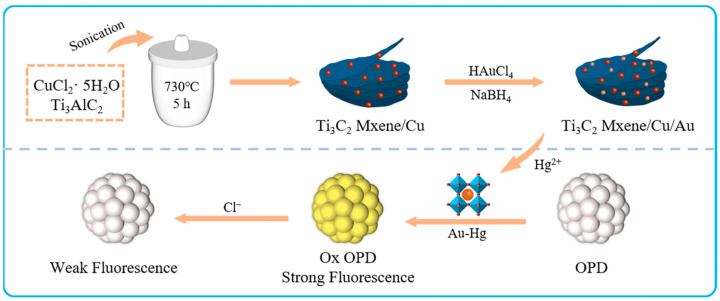
Schematic illustration of synthesis of Au/Cu/Ti_3_C_2_ NSs and the recognition of Hg^2+^ and Cl^−^.

Herein, we fabricated a smartphone-enabled fluorescence and colorimetric platform for the on-site detection of Hg^2+^ and Cl^−^ based on the oxidase-like activity of Au/Cu/Ti_3_C_2_ NSs (Figure 1). The Au nanoparticles (NPs) were in-situ loaded onto the surface of Cu/Ti_3_C_2_ NSs to form Au/Cu/Ti_3_C_2_ NSs. The Au/Cu/Ti_3_C_2_ NSs were characterized and their enzyme-like activities were studied. After the addition of Hg^2+^ with different concentrations, the formation of Hg–Au alloys could promote the oxidization of o-phenylenediamine (OPD) to generate a new fluorescence emission peak at 570 nm. Therefore, a turn-on fluorescence method for the detection of Hg^2+^ could be established. Given the interaction between Hg^2+^ and Cl^−^ and the inhibition of the formation of Hg–Au alloys, the fluorescence intensity was constantly quenched to achieve the quantitative detection of Cl^−^. Depending on the color change with the detection of Hg^2+^ and Cl^−^, a convenient colorimetric method for Hg^2+^ and Cl^−^ could be developed by using digital images combined with smartphones (color recognizers) to analyze RGB values in concentrations of Hg^2+^ or Cl^−^. The digital picture sensor based on a smartphone tested Cl^−^ and Hg^2+^ in water samples and held great promise for environmental monitoring.

## 2. Results and Discussion

### 2.1. Characterization of Au/Cu/Ti_3_C_2_ NSs

**Figure 2:**
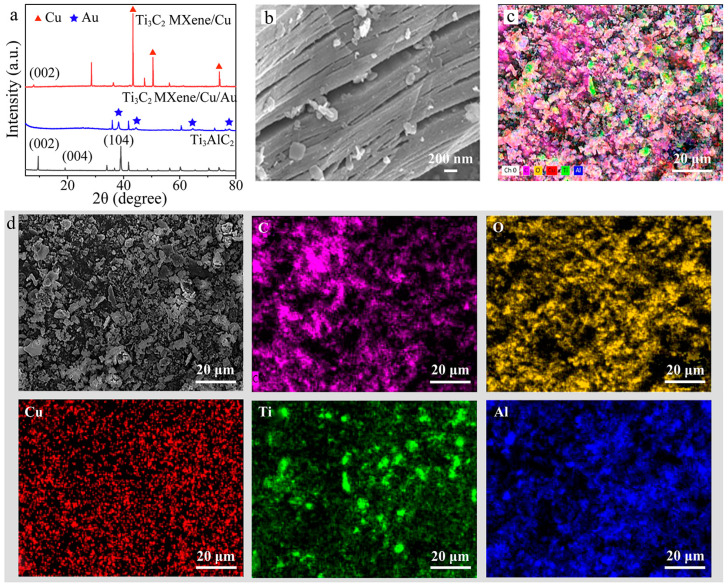
(**a**) XRD patterns of Ti_3_AlC_2_, Cu/Ti_3_C_2_ NSs, and Au/Cu/Ti_3_C_2_ NSs, (**b**) SEM patterns of Cu/Ti_3_C_2_ NSs, (**c**) EDS element analysis of multilayer Cu/Ti_3_C_2_ NSs, and (**d**) the element distribution map.

Cu/Ti_3_C_2_ NSs was prepared by the one-step Lewis acidic etching route. Ti_3_AlC_2_ and CuCl_2_ were mixed evenly in a mortar and grinded for 10 min. The pulverized mixture was transferred to a porcelain crucible and roasted at 730 °C for 1 h. After cooling and quenching, FeCl_3_ solution and deionized water were added to wash the powder. The power was collected and dried in vacuum at 70 °C to obtain the products. The synthesized multilayer Cu/Ti_3_C_2_ NSs and Au/Cu/Ti_3_C_2_ NSs were characterized by SEM, TEM, XPS, and XRD. Figure 2a showed the XRD patterns of Ti_3_AlC_2_, Cu/Ti_3_C_2_ NSs, and Au/Cu/Ti_3_C_2_ NSs. Compared with the pristine Ti_3_AlC_2_, most diffraction peaks disappeared in the final Cu/Ti_3_C_2_ NSs. There were several wide low diffraction peaks in the 2θ ranging from 5° to 80°, which indicated that Ti_3_AlC_2_ had been successfully exfoliated into layered Cu/Ti_3_C_2_ NSs. The corresponding diffraction peak of Ti_3_C_2_ (002) shifted from 9.63° to 7.98°, demonstrating that the interlayer spacing had been expanded. The diffraction peaks at 2θ of 43.30°, 50.46°, and 74.09° corresponded to Cu, while those at 2θ of 38.1°, 44.5°, 65.1°, and 77.6° corresponded to Au, which demonstrated the successful loading of Au on the surface of Cu/Ti_3_C_2_ NSs [35,36]. Figure 2b shows the SEM image of multilayer Cu/Ti_3_C_2_ NSs, revealing that Cu/Ti_3_C_2_ NSs material had a good layered microstructure, which was consistent with the XRD data analysis and similar to what was previously reported for MXenes and obtained through etching methods with the HF- or F-containing electrolyte. Figure 2c,d shows EDS diagrams of multilayer Cu/Ti_3_C_2_ NSs. The element distribution map of Cu/Ti_3_C_2_ NSs showed that the nanosheets contained C, O, Cu, Ti, and a small amount of Al, which demonstrated that the majority of Al was etched by this method.

**Figure 3:**
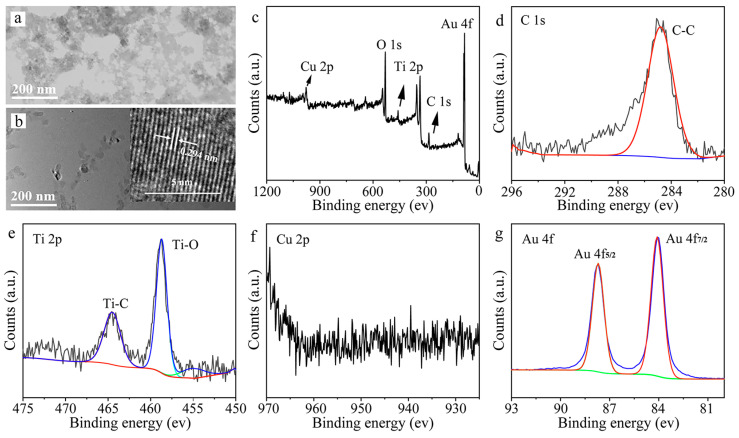
(**a**) TEM image of Cu/Ti_3_C_2_ NSs, (**b**) TEM image of Au/Cu/Ti_3_C_2_ NSs, the inset of (**b**) was the HRTEM of Au/Cu/Ti_3_C_2_ NSs, (**c**) survey XPS spectra of Au/Cu/Ti_3_C_2_ NSs and spectra of Ti_3_C_2_ MXene/Cu/Au NSs, (**d**) C 1s, (**e**) Ti 2p, (**f**) Cu 2p, and (**g**) Au 4f.

The TEM images of Cu/Ti_3_C_2_ NSs and Au/Cu/Ti_3_C_2_ NSs clearly demonstrated that Cu nanoparticles and Au nanoparticles were uniformly distributed on the surface of Ti_3_C_2_ NSs (Figure 3a,b). The HRTEM image in the upper right corner of Figure 3b showed a lattice spacing of 0.29 nm, which corresponds to the (111) crystal plane of Au. The elemental composition and chemical bond of Au/Cu/Ti_3_C_2_ NSs were characterized by XPS spectra (Figure 3c). Au/Cu/Ti_3_C_2_ NSs contained five characteristic peaks according to the full spectrum analysis of XPS: Cu 2p (977.6 eV), O 1s (530.9 eV), Ti 2p (458.9 eV), C 1s (284.5 eV), and Au 4f (85.5 eV). It proved that Au/Cu/Ti_3_C_2_ NSs was successfully synthesized. The chemical bonds of Au/Cu/Ti_3_C_2_ NSs were analyzed by high-resolution XPS spectra, including Cu 2p, Ti 2p, C 1s, and Au 4f. As shown in Figure 3d–g, a deconvolution peak corresponding to C-C existed in the high-resolution XPS spectrum of C 1s. The high-resolution XPS spectrum of Ti 2p contained two deconvolution peaks corresponding to Ti-C and Ti-O. However, there were two deconvolution peaks in the high-resolution XPS spectra of Au 4f, indicating that Au mainly existed in the form of 0 valence and 1 valence.

### 2.2. Oxidase-like Activity of Au/Cu/Ti_3_C_2_ NSs

**Figure 4:**
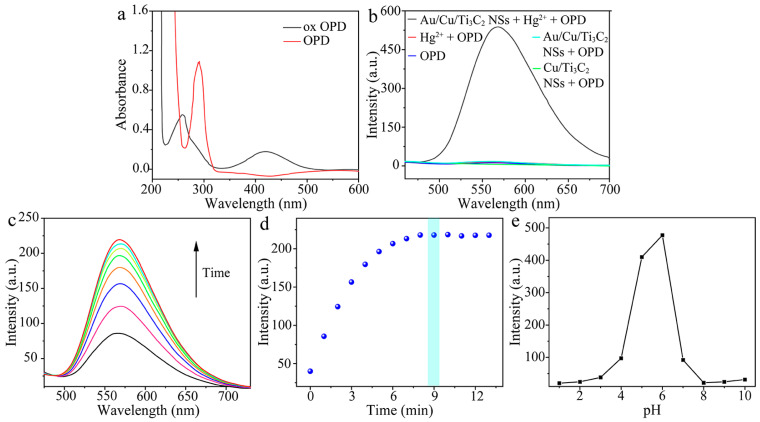
(**a**) UV-vis spectra of OPD and DAP, (**b**) the feasibility of catalytic system for the detection of Hg^2+^ using florescence spectra (Au/Cu/Ti_3_C_2_ NSs + Hg^2+^ + OPD, Hg^2+^ + OPD, Au/Cu/Ti_3_C_2_ NSs + OPD, OPD, Cu/Ti_3_C_2_ NSs + OPD), (**c**) fluorescence spectra of DAP with time change, (**d**) scatter plot of fluorescence intensity of DAP and incubation time (*n* = 3), and (**e**) the influence of pH on fluorescence of the DAP (*n* = 3).

The feasibility of Au/Cu/Ti_3_C_2_ NSs + Hg^2+^ to catalysis substrate OPD was investigated by absorption spectra (Figure 4a) and fluorescence spectra (Figure 4b). In the absorption spectra, a new absorption peak at 420 nm appeared after the oxidation of OPD to 2,3-diaminopenazine (DAP) with the catalysis of Au/Cu/Ti_3_C_2_ NSs + Hg^2+^. In the fluorescence spectra, only when Au/Cu/Ti_3_C_2_ NSs and Hg^2+^ co-exist, OPD could be rapidly oxidized and a new fluorescence emission peak of DAP was generated at 570 nm. The oxidization process of OPD by dissolved oxygen was slow. The addition of Hg^2+^ to the solution resulted in the formation of Hg–Au alloys on the surface of Au/Cu/Ti_3_C_2_ NSs, which could accelerate the oxidization of OPD to generate DAP with both new absorption and a fluorescence signal [37]. Therefore, we intended to develop an absorption and fluorescent method for the sensing of Hg^2+^ via this phenomenon. To achieve the sensitive detection of Hg^2+^, several enzymatic factors that may influence the enzyme-substrate interactions were studied. The reaction time dependence of the Hg^2+^-induced fluorescence of DAP increasing was investigated. The fluorescence intensity increased when the reaction time was from 0 to 9 min and then kept stable with the reaction time from 9 to 12 min (Figure 4c,d). Therefore, 9 min was chosen as the optimal reaction time. The pH dependence of the Hg^2+^-induced fluorescence of DAP increasing was investigated in the pH range of 1.0 to 10.0. The fluorescence intensity increased abruptly from pH 1.0 to 6.0, and then decreased from pH 6.0 to 10.0 (Figure 4e). Therefore, a pH of 6.0 was chosen as the optimal pH.

### 2.3. Detection of Hg^2+^

**Figure 5:**
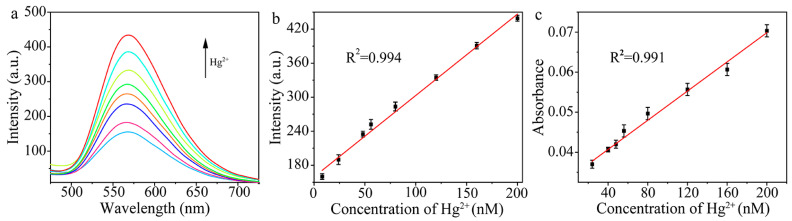
(**a**) Fluorescence spectra of the system with different concentrations of Hg^2+^, (**b**) linear relationship between fluorescence intensity and Hg^2+^ concentration (*n* = 3), and (**c**) linear relationship between absorbance and Hg^2+^ concentration (*n* = 3).

The feasibility of this method for the detection of Hg^2+^ was verified. Au/Cu/Ti_3_C_2_ NSs reacted with different concentrations of Hg^2+^ for 9 min to obtain the oxidase-like Au–Hg alloy, which catalyzed the oxidation of OPD to produce DAP. As shown in Figure 5a, when the concentration of Hg^2+^ gradually increased in the range from 0 to 200 nM, the fluorescence emission at 570 nm from the catalytic product DAP also increased continuously. Therefore, we established the fluorescence method for the detection of Hg^2+^. The linear relationship between the fluorescence intensity of DAP and the Hg^2+^ concentration was I = 1.434C + 159.575 (C is the concentration of Hg^2+^, nM). The linearly range for the detection of Hg^2+^ was 8.0 to 200.0 nM, with the detection limit of 0.8 nM. Since the new absorption peak of DAP at 420 nm also enhanced with the increased concentration of Hg^2+^, the correlation between the absorbance and concentration of Hg^2+^ was analyzed, as shown in Figure 5c. Therefore, we also established the colorimetric method for the detection of Hg^2+^. Within the concentration range of 24.0 to 200.0 nM, the linear equation of absorbance and Hg^2+^ concentration was A = 1.82 × 10^−4^C + 0.0334 (C is the concentration of Hg^2+^, nM), and the detection limit was 2.4 nM.

### 2.4. Detection of Cl^−^

**Figure 6:**
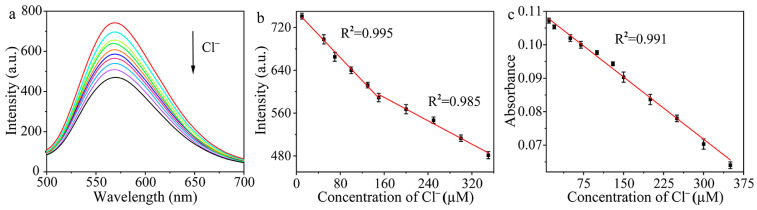
(**a**) Fluorescence spectra of the system with different concentrations of Cl^−^, (**b**) linear relationship between fluorescence intensity and Cl^−^ concentration (*n* = 3), and (**c**) linear relationship between absorbance and Cl^−^ concentration (*n* = 3).

To study whether the Cl^−^ can be detected via the influence on the oxidase-like activity of Hg–Au alloys on the surface of Au/Cu/Ti_3_C_2_ NSs, a series of Cl^−^ with concentrations in the range from 0 to 350 μM was added to the system Au/Cu/Ti_3_C_2_ NSs + Hg^2+^ (Figure 6). Figure 6a showed that as the concentration of Cl^−^ increased, the fluorescence intensity of DAP decreased gradually. As shown in Figure 6b, there were two linear relationships between the fluorescence intensity of DAP and Cl^−^ concentration. Therefore, we established the fluorescence method for the detection of Cl^−^. When Cl^−^ concentration increased from 10.0 μM to 150.0 μM, the linear equation of fluorescence intensity and Cl^−^ concentration was I = −1.076C + 749.776 (C is the concentration of Cl^−^, μM). When the concentration of Cl^−^ increased gradually from 150.0 μM to 350.0 μM, the linear equation of fluorescence intensity and Cl^−^ concentration was I = −0.546C + 676.657 (C is the concentration of Cl^−^, μM). The detection limit was 27.0 nM. In addition, the relationship between the absorbance and the added Cl^−^ concentration was also explored, as shown in Figure 6c. Therefore, we also established the colorimetric method for the detection of Cl^−^. In the range of 0.0 to 350.0 μM, the absorbance of the system had a good response to Cl^−^ concentration. The linear equation was A = −1.24 × 10^−4^C + 0.109 (C is the concentration of Cl^−^, μM), and the detection limit was 1.0 μM.

### 2.5. POCT for Hg^2+^ and Cl^−^

**Figure 7:**
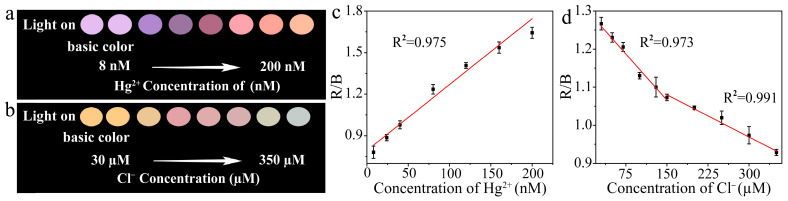
(**a**) Pictures taken under the irradiation of 365 nm UV lamp when different concentrations of Hg^2+^ were added, (**b**) pictures taken under the irradiation of 365 nm UV lamp when different concentrations of Cl^−^ were added, (**c**) linear relationship between R/B value and Hg^2+^ concentration (*n* = 3), and (**d**) linear relationship between R/B value and Cl^−^ concentration (*n* = 3).

In order to simplify the detection procedure and realize instrument-free detection, portable test strips were prepared for the detection of Hg^2+^ and Cl^−^. The smartphone-assist platform was established and consisted of test strips containing Au/Cu/Ti_3_C_2_ NSs and a smartphone with an app as the signal reader and analyzer for the test strips. As the concentration of Hg^2+^ increased, the fluorescence color of the test strips changed from purple to pink (Figure 7a). Figure 7b showed the fluorescence color picture of the test strips with different concentrations of Cl^−^, and with the continuous addition of Cl^−^ with different concentrations, the fluorescence color of the test strips changed from yellow to light green. The app software (color recognizer) in the smartphone acquired the color parameters (R, G, B value) of the photos. The RGB ratio (R/B) had a linear relationship with the concentration of Hg^2+^ over the range of 8.0 to 200.0 nM (Figure 7c). The linear equation of R/B and Hg^2+^ concentration was R/B = 0.0048C + 0.79 (C is the concentration of Hg^2+^, nM). The detection limit was 0.8 nM. There are two linear relationships between R/B and Cl^−^ concentration, as shown in Figure 7d. Within the concentration range of 30.0 to 150.0 μM and 150.0 to 350.0 μM, the linear equations were R/B = −0.0016C + 1.309 and R/B = −7.396 × 10^−4^C + 1.191 (C is the concentration of Cl^−^, μM), and the detection limit was 3.0 μM. Compared with the fluorescence and colorimetric methods, the digital image method based on Au/Cu/Ti_3_C_2_ NSs was expected to be applied to the real-time and rapid detection of Hg^2+^ and Cl^−^ concentrations in the field.

### 2.6. Selectivity for Hg^2+^ and Cl^−^

**Figure 8:**
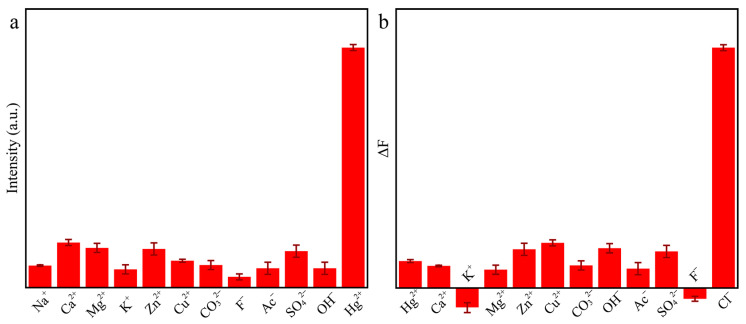
The selectivity of the catalytic system to (**a**) Hg^2+^ and (**b**) Cl^−^ (*n* = 3).

Selectivity is an important factor to evaluate the practicality of the method. In order to explore the practicability of this system for the detection of Hg^2+^, different anions and cations, including Na^+^, Ca^2+^, Mg^2+^, K^+^, Zn^2+^, Cu^2+^, CO_3_^2−^, F^−^, Ac^−^, SO_4_^2−^, and OH^−^ were added to the Au/Cu/Ti_3_C_2_ NSs + OPD system. As shown in Figure 8a, the Au/Cu/Ti_3_C_2_ NSs + OPD system had an obvious response to Hg^2+^, while the fluorescence intensity of the system did not change significantly when detecting other ions.

In addition, the reliability of the Ti_3_C_2_ MXene/Cu/Au + Hg^2+^ + OPD system for the detection of Cl^−^ concentration was also discussed. Different anions and cations were added to the Ti_3_C_2_ MXene/Cu/Au + Hg^2+^ + OPD system, and the fluorescence intensity of the detection system was recorded by a fluorescence spectrometer. As shown in Figure 8b, the Ti_3_C_2_ MXene/Cu/Au + Hg^2+^ + OPD system had good specificity for the analysis of Cl^−^, while there was no obvious change in fluorescence intensity when detecting other ions. Therefore, it can be concluded that the detection system has good selectivity for Hg^2+^ and Cl^−^. Meanwhile, the influence of Cl^−^ concentration on the detection of mercury ions was studied (Figure S1). When the concentration of Cl^−^ ranged from 0 to 500 μM, there was no obvious effect on the detection of mercury ions.

### 2.7. Real Sample Detection

In order to verify the applicability of this method in detecting Hg^2+^ concentration in actual samples, water from Shahu Lake, East Lake, and Yangtze River was selected as actual samples, and the content of Hg^2+^ was evaluated using test strips. The specific results are shown in Table 1. The recoveries of Hg^2+^ concentration was 93.3 to 109.7%, and RSD ranged from 1.4% to 5.5%. The real sample detection for chloride ions was also investigated. As shown in Table S1, the recoveries of Cl^−^ concentration vary from 93.3 to 109.7% with the RSDs of recoveries less than 5.5%. The results demonstrate that the developed method is practical and reliable for the detection of Hg^2^ and Cl^−^.

## 3. Experimental

### 3.1. Materials

All reagents were of at least analytical grade and used as received without purification. Ti_3_AlC_2_ was acquired from Forsman Technology Co., Ltd. (Beijing, China). Hg(NO_3_)_2_, CuSO_4_·5H_2_O, and HAuCl_4_ were supplied by Macklin Biochemical Co., Ltd. (Shanghai, China). O-Phenylenediamine (OPD) was purchased from Aladdin Biochemical Technology Co., Ltd. (Shanghai, China). NaBH_4_, NaCl, Na_2_CO_3_, FeCl_3_, MgCl_2_·6H_2_O, ZnCl_2_, KCl, CaCl_2_, Na_2_SO_4_, and KNO_3_ were provided by Sinopharm Chemical Reagent Co., Ltd. (Shanghai, China). All solutions were freshly prepared before use.

### 3.2. Instuments

The UV-vis absorption measurements were observed by a Lambda 35 UV analyzer (Perkin-Elmer, Waltham, MA, USA). The excitation and emission spectra were obtained on a LS55 spectrophotometer (Perkin-Elmer, Waltham, MA, USA). The transmission electron microscopy (TEM) was performed using a TecnaiG20 transmission electron microscope (FEI, Hillsboro, OR, USA) and LEPL-Model 2100 F instrument. High-resolution transmission electron microscopy (HRTEM) was measured by a JEM-2100 UHR (JEOL, Tokyo, Japan). X-ray photo electron spectroscopy (XPS) was performed with a VGEscalab 200 spectrometer using an aluminum anode (AlKα) operating at 510 W with a background pressure of 2 × 10^−9^ mbar (Escalab, Waltham, MA, USA).

### 3.3. Preparation of Au/Cu/Ti_3_C_2_ NSs

Cu/Ti_3_C_2_ NSs was synthesized by a one-step Lewis acidic etching route. After being placed in a mortar, 0.5 g of Ti_3_AlC_2_ and 2.6 g of CuCl_2_·2H_2_O were mixed evenly and ground for 10 min. The pulverized mixture was transferred to a 50 mL porcelain crucible and roasted at 730 °C for 1 h. After cooling and quenching, 50.0 mL of 3.0 M FeCl_3_ solution was added to the powder and stirred continuously for 3 h. The precipitate was washed with deionized water, collected, and dried for 12 h in vacuum at 70 °C.

Then, 100.0 mg of Cu/Ti_3_C_2_ NSs was dispersed in 25.0 mL of deionized water and ultrasonicated for 5 h. The suspension with Cu/Ti_3_C_2_ NSs was gently collected with centrifugation at 5000 r for 8 min. Then the suspension was further centrifuged at 9500 r for 30 min and the collected precipitates were dispersed in 5 mL of deionized water for future use.

Subsequently, 500 µL of 0.5 mol/L HAuCl_4_ was added to the above solution, followed by 250 µL of 0.1 M NaBH_4_ quickly, and stirred for 1 h. Then, the precipitates of Au/Cu/Ti_3_C_2_ NSs were collected by centrifugation at 9500 r for 30 min and dispersed in 6 mL of deionized water for future use.

### 3.4. Fluorescence and Colorimetric Method for the Detection of Hg^2+^ and Cl^−^

The method for the detection of Hg^2+^ is described below. First, 60 µL of Au/Cu/Ti_3_C_2_ NSs and 30 µL of Hg^2+^ with different concentrations were successively added into 880 µL of deionized water. The Au–Hg alloys with oxidase activity were obtained after being incubated for 5 min. Finally, 30 µL of 0.1 M OPD was added into the mixture. The concentration of Hg^2+^ was monitored by fluorescence and colorimetric signals after incubation at 37 °C for 9 min.

The method for the detection of Cl^−^ is described below. First, 60 µL of Au/Cu/Ti_3_C_2_ NSs, 50 µL of 8 µM Hg^2+^, and 20 µL of Cl^−^ with different concentrations were successively added to 840 µL of deionized water. Then, 30 µL of 0.1 M OPD was added into the mixture after incubation for 5 min. The concentration of Cl^−^ was monitored by fluorescence colorimetric signals after incubation at 37 °C for 9 min.

### 3.5. Preparation of Test Papers

Commercial fiber filter paper was used to make test paper with a diameter of 1 cm. The fiber filter paper was immersed in Au/Cu/Ti_3_C_2_ NSs solution for 10 min and then dried in an oven at 60 °C to obtain the test paper. Afterwards, 30 µL of 0.1 M OPD and 30 µL of Hg^2+^ with different concentrations were successively dropped on the test paper. Then, the colorimetric signals were recorded by a Lambda 35 UV analyzer. The colorimetric images were collected with a smartphone under 365 nm ultraviolet light. The test paper for Cl^−^ was prepared with the same process with test paper for Hg^2+^.

### 3.6. Real Sample Analysis

The potential application of the proposed method was demonstrated by applying it to detecting Hg^2+^ and Cl^−^ in water samples. All the water as the real sample was taken from a nearby lake and river in Wuhan (China). The water from East Lake, Shahu Lake, and Yangtze River was selected as the research objects. The samples were filtered by 0.22 μm microporous membrane and diluted to 20 times as the real samples to be tested.

## 4. Conclusions

In this work, an efficient fluorescence and colorimetric method with the favor of a smartphone for the on-site detection of Hg^2+^ and Cl^−^ was fabricated based on Au/Cu/Ti_3_C_2_ NSs. Characterization techniques confirmed the sucessful synthesis of Au/Cu/Ti_3_C_2_ NSs. Because of the formation of the Au–Hg alloy with oxidase-like activity, the OPD was transferred into ADP with a fluorescence emission at 570 nm and an obvious color change. Therefore, a turn-on fluorescence and colorimetric method for the detection of Hg^2+^ was established. As the addition of Cl^−^, the fluorescence intensity was constantly quenched to achieve the continuous quantitative detection of Cl^−^. A series of color variations of the paper strip can be observed by the naked eye and digital images with the introduction of different concentrations of Hg^2+^ or Cl^−^. The smartphone with the app (color recognizers) could favor the digital images of Hg^2+^ or Cl^−^. The Au/Cu/Ti_3_C_2_ NSs-based platform displayed potent sensitivity, broad applicability, favorable selectivity, and significant simplicity. Our proposed Au/Cu/Ti_3_C_2_ NSs-based method is a promising method for environmental monitoring.

## Figures and Tables

**Table 1:** Analytical results for detection of Hg^2+^ in real samples (*n* = 3).

Samples	Spiked (µM)	Found (µM)	Recovery (%)	RSD (%)
Shahu Lake	24	25.23	105.1	2.3
	80	87.78	109.7	2.1
	160	149.22	93.3	2.5
East Lake	24	23.04	96.0	2.8
	80	82.29	102.9	5.5
	160	159.1	99.4	1.4
Yangtze River	24	22.49	93.7	2.6
	80	83.39	104.3	3.1
	160	154.71	96.7	4.5

## Data Availability

Not applicable.

## Supplementary Materials

The following supporting information can be downloaded at: https://www.mdpi.com/article/10.3390/molecules28145355/s1. Figure S1: The influence of Cl^−^ concentration on the detection of mercury ions (*n* = 3, the concentration of Hg^2+^ is 40 nM); Table S1: Analytical results for the detection of Cl^−^ in real samples (*n* = 3).

## Sample Availability

Samples of the compounds are available from the authors.
