# Supplementary material for: Smartphone-Enabled Fluorescence and Colorimetric Platform for the On-Site Detection of Hg2+ and Cl− Based on the Au/Cu/Ti3C2 Nanosheets
Source: Molecules. 2023 Jul 12;28(14):5355. doi: 10.3390/molecules28145355 (PMC10386442; doi:10.3390/molecules28145355)
Supplement: Supplementary file 1 [file molecules-28-05355-s001.zip › molecules-2473904-supplementary.pdf]

Supplementary Material

Smartphone-Enabled Fluorescence and Colorimetric Platform for the On-Site Detection of Hg^{2+} and Cl^- Based on the $Au/Cu/Ti_3C_2$ Nanosheets

Keyan Chen [1], Shiqi Fu [1], Chenyu Jin [1], Fan Guo [1], Yu He [2], Qi Ren [1,*] and Xuesheng Wang [1,*]

[1] School of Public Health, North China University of Science and Technology, Tangshan 063210, China; kychen1107@163.com (K.C.); fushiqi17_f@163.com (S.F.); jincy2021@126.com (C.J.); gf0518@126.com (F.G.)
[2] Ministry of Education Key Laboratory for the Synthesis and Application of Organic Functional Molecules, College of Chemistry and Chemical Engineering, Hubei University, Wuhan 430062, China; heyu@hubu.edu.cn
* Correspondence: renqi@ncst.edu.cn (Q.R.); xswang64@163.com (X.W.)

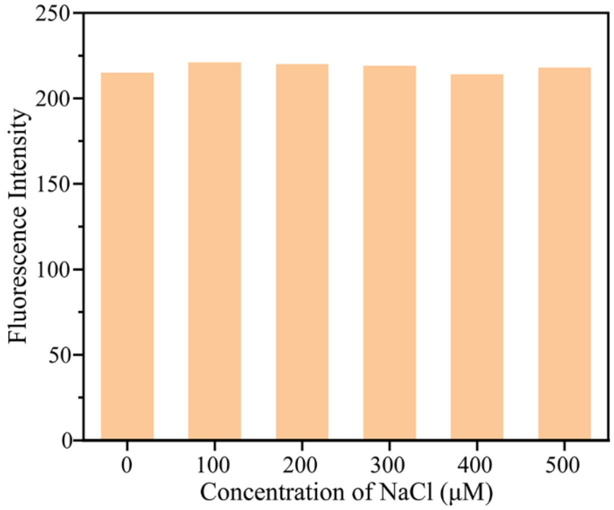

Figure S1. The influence of Cl^- concentration on the detection of mercury ions. (n = 3, the concentration of Hg^{2+} is 40 nM).

Table S1. Analytical results for detection of Cl^- in real samples (n = 3)

Sample	Spiked (μM)	Found (μM)	Recovery (%)	RSD (%)
Shahu Lake	20	18.97	94.85	2.8
	80	76.83	96.04	5.0
	150	148.3	98.87	4.3
East Lake	20	21.32	106.6	3.4
	80	83.44	104.3	3.6
	150	154.3	102.9	3.5
Yangtze River	20	18.05	90.25	4.1
	80	77.88	97.35	3.3
	150	143.6	95.73	4.3
